# The genome sequence of the black needle fly, *Leuctra nigra* (Olivier, 1811)

**DOI:** 10.12688/wellcomeopenres.19082.1

**Published:** 2023-02-22

**Authors:** Caleala Clifford, Natural History Museum Genome Acquisition Lab, Darwin Tree of Life Barcoding collective, Wellcome Sanger Institute Tree of Life programme, Wellcome Sanger Institute Scientific Operations: DNA Pipelines collective, Tree of Life Core Informatics collective, Craig R. Macadam, Benjamin W. Price, Darwin Tree of Life Consortium

**Affiliations:** 1 Natural Resources Wales, Cardiff, Wales, UK; 2 Buglife – The Invertebrate Conservation Trust, Stirling, UK; 3 Life Science Department,, Natural History Museum, London, UK

**Keywords:** Leuctra nigra, stonefly, genome sequence, chromosomal, Plecoptera

## Abstract

We present a genome assembly from an individual male *Leuctra nigra* (black needle fly; Arthropoda; Insecta; Plecoptera; Leuctridae). The genome sequence is 536.3 megabases in span. Most of the assembly is scaffolded into 13 chromosomal pseudomolecules *, *including the X sex chromosome. The mitochondrial genome has also been assembled and is 17.6 kilobases in length.

## Species taxonomy

Eukaryota; Metazoa; Ecdysozoa; Arthropoda; Hexapoda; Insecta; Pterygota; Neoptera; Polyneoptera; Plecoptera; Nemouroidea; Leuctridae; Leuctrinae; *Leuctra*; *Leuctra nigra* (Olivier, 1811) (NCBI:txid143735).

## Background

**Figure 1.**
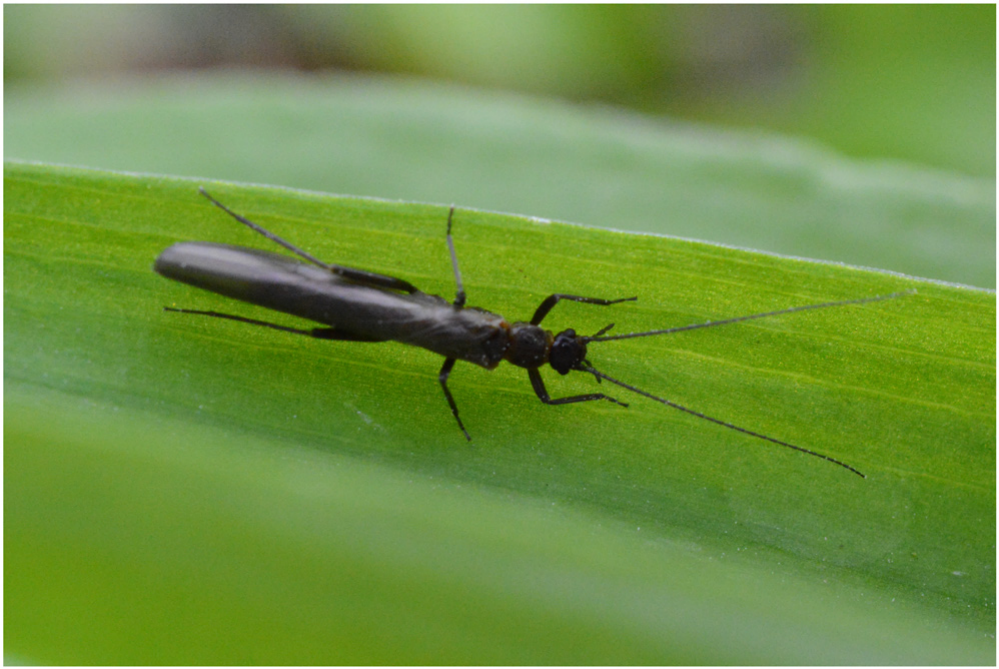
*Leuctra nigra* adult (c) Wolfram Graf.JPG.

The stonefly *Leuctra nigra* ( Figure 1) is a western Palearctic species found across central Europe from France to Ukraine and north to Fennoscandia. It is found throughout Britain and Ireland, generally in northern and western areas, although there are scattered records from south-east England and the south of Ireland.

It is considered a eurytherm ( Ravizza & Vinçon, 1998) and is typically found in low densities in silty, sandy and fine gravel habitats, but also amongst woody debris, twigs roots and logs ( Baars & Kelly-Quinn, 2006; O’Connor and Costello, 1997). Some of these streams can be very small and easily overlooked. This species is known to predominate in acidified moorland and coniferous forest streams in both Denmark and Great Britain ( Friberg *et al.*, 1998; Thomsen & Friberg, 2002; Stoner *et al.*, 1984; Murphy *et al.,* 2013). In Ireland, *L. nigra* is much rarer although it has been recorded in episodically acidic streams ( Feeley, 2012; Feeley & Kelly-Quinn, 2014). Adults are generally also found in low densities amongst vegetation nearby associated streams ( O’Connor & Costello, 1997).

The life cycle of *Leuctra nigra* is highly variable across Europe. Several studies have reported a one year, univoltine, life cycle ( Brinck, 1949; Hynes, 1941). However, studies have found that larvae take two years to develop in northern England and Denmark ( Elliott, 1987; Thomsen & Friberg, 2002), or a mixture of both one and two years in acidic and iron-rich watercourses ( Hildrew *et al.*, 1980). Larvae are opportunistic feeders utilising a range of allochthonous and autochthonous plant material ( Graf *et al.*, 2002; Graf *et al.*, 2009; Henderson *et al.*, 1990; Lillehammer, 1988; Thomsen & Friberg, 2002).

The high-quality genome sequence described here is, to our knowledge, the first reported for *Leuctra nigra*, and has been generated as part of the Darwin Tree of Life project. It will aid in understanding the biology, physiology and ecology of the species.

## Genome sequence report

The genome was sequenced from one male *Leuctra nigra* collected from River Taff Fawr, Garwnant, UK (latitude 51.808259, longitude –3.44498). A total of 32-fold coverage in Pacific Biosciences single-molecule HiFi long reads was generated. Primary assembly contigs were scaffolded with chromosome conformation Hi-C data. Manual assembly curation corrected 458 missing joins or mis-joins and removed seven haplotypic duplications, reducing the scaffold number by 47.9%, and increasing the scaffold N50 by 4.62%.

**Figure 2.**
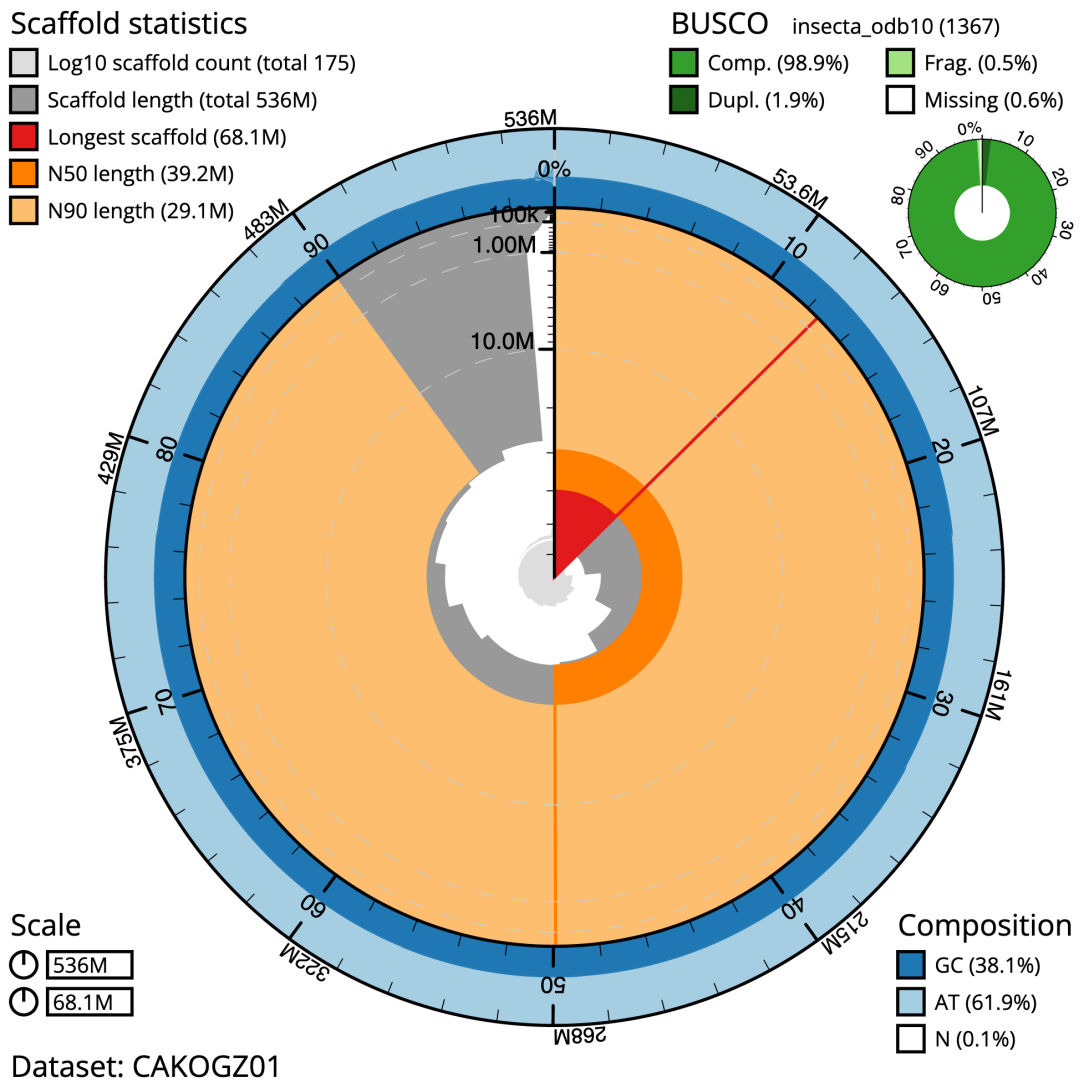
Genome assembly of *Leuctra nigra*, ipLeuNigr2.1: metrics. The BlobToolKit Snailplot shows N50 metrics and BUSCO gene completeness. The main plot is divided into 1,000 size-ordered bins around the circumference with each bin representing 0.1% of the 536,298,261 bp assembly. The distribution of scaffold lengths is shown in dark grey with the plot radius scaled to the longest scaffold present in the assembly (68,080,570 bp, shown in red). Orange and pale-orange arcs show the N50 and N90 scaffold lengths (39,220,573 and 29,107,315 bp), respectively. The pale grey spiral shows the cumulative scaffold count on a log scale with white scale lines showing successive orders of magnitude. The blue and pale-blue area around the outside of the plot shows the distribution of GC, AT and N percentages in the same bins as the inner plot. A summary of complete, fragmented, duplicated and missing BUSCO genes in the insecta_odb10 set is shown in the top right. An interactive version of this figure is available at https://blobtoolkit.genomehubs.org/view/ipLeuNigr2.1/dataset/CAKOGZ01/snail.

The final assembly has a total length of 536.3 Mb in 174 sequence scaffolds with a scaffold N50 of 39.2 Mb ( Table 1). Most (98.52%) of the assembly sequence was assigned to 13 chromosomal-level scaffolds, representing 12 autosomes and the X sex chromosome. The X chromosome was found at half coverage and no Y chromosome was found. Chromosome-scale scaffolds confirmed by the Hi-C data are named in order of size ( Figure 2– Figure 5; Table 2). The assembly has a BUSCO v5.3.2 ( Manni *et al.*, 2021) completeness of 98.9% (single 97%, duplicated 1.9%) using the insecta_odb10 reference set. While not fully phased, the assembly deposited is of one haplotype. Contigs corresponding to the second haplotype have also been deposited.

**Table 1.** Genome data for *Leuctra nigra*, ipLeuNigr2.1.

Project accession data		
Assembly identifier	ipLeuNigr2.1	
Species	*Leuctra nigra*	
Specimen	ipLeuNigr2	
NCBI taxonomy ID	143735	
BioProject	PRJEB50932	
BioSample ID	PRJEB50932	
Isolate information	ipLeuNigr2 (PacBio); ipLeuNigr1 (Hi-C)	
Assembly metrics*		*Benchmark*
Consensus quality (QV)	57.2	*≥ 50*
*k*-mer completeness	99.99%	*≥ 95%*
BUSCO**	C:98.9%[S:97.0%,D:1.9%], F:0.5%,M:0.6%,n:1,367	*C ≥ 95%*
Percentage of assembly mapped to chromosomes	98.52%	*≥ 95%*
Sex chromosomes	X chromosome	*localised homologous pairs*
Organelles	Mitochondrial genome assembled	*complete single alleles*
Raw data accessions		
PacificBiosciences SEQUEL II	ERR8705875	
Hi-C Illumina	ERR8702800	
Genome assembly		
Assembly accession	GCA_934045905.1	
*Accession of alternate haplotype*	GCA_934046545.1	
Span (Mb)	536.3	
Number of contigs	1,718	
Contig N50 length (Mb)	0.6	
Number of scaffolds	174	
Scaffold N50 length (Mb)	39.2	
Longest scaffold (Mb)	68.1	

* Assembly metric benchmarks are adapted from column VGP-2020 of “Table 1: Proposed standards and metrics for defining genome assembly quality” from ( Rhie *et al.*, 2021). ** BUSCO scores based on the insecta_odb10 BUSCO set using v5.3.2. C = complete [S = single copy, D = duplicated], F = fragmented, M = missing, n = number of orthologues in comparison. A full set of BUSCO scores is available at https://blobtoolkit.genomehubs.org/view/ipLeuNigr2.1/dataset/CAKOGZ01/busco.

**Figure 3.**
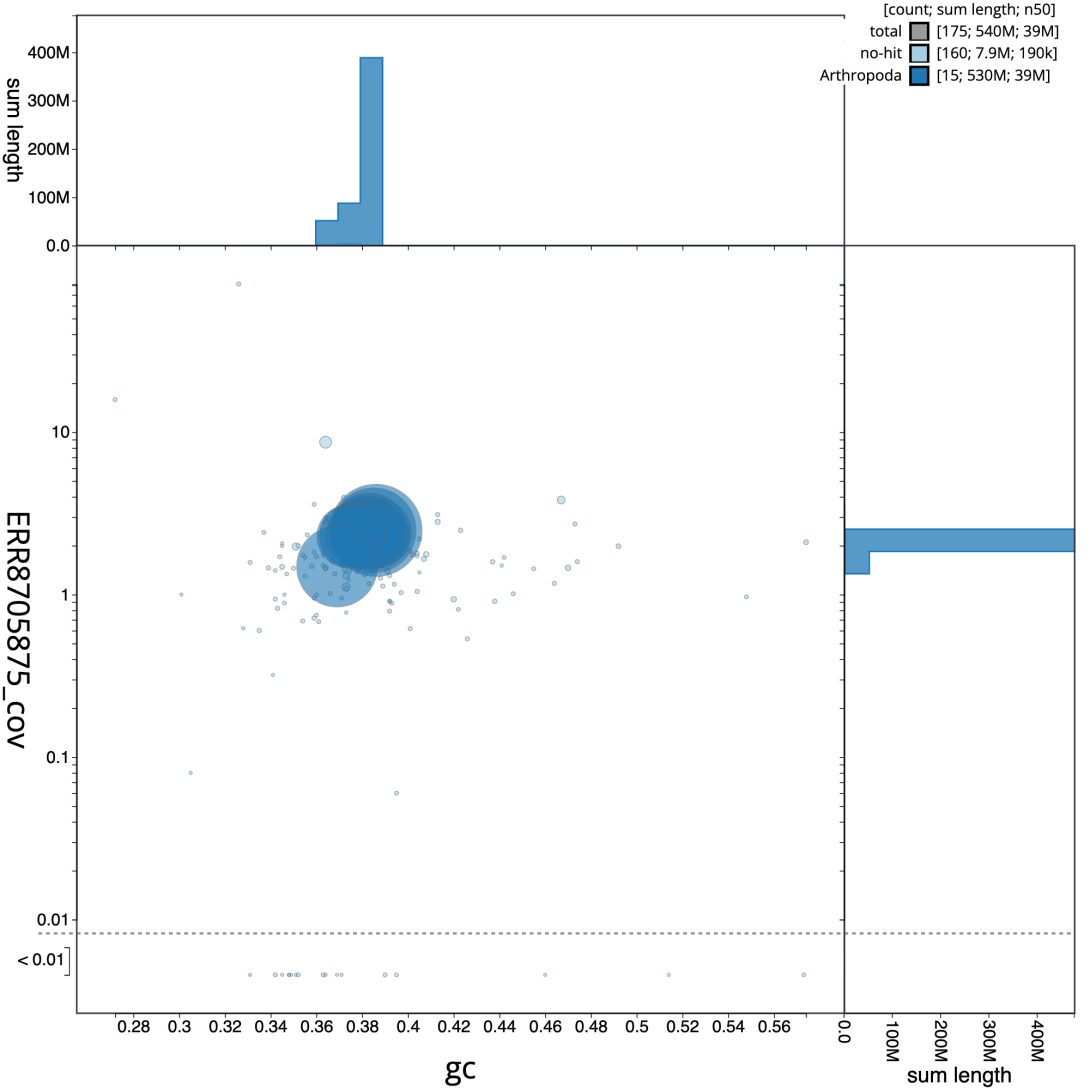
Genome assembly of *Leuctra nigra*, ipLeuNigr2.1: GC coverage. BlobToolKit GC-coverage plot. Scaffolds are coloured by phylum. Circles are sized in proportion to scaffold length. Histograms show the distribution of scaffold length sum along each axis. An interactive version of this figure is available at https://blobtoolkit.genomehubs.org/view/ipLeuNigr2.1/dataset/CAKOGZ01/blob.

**Figure 4.**
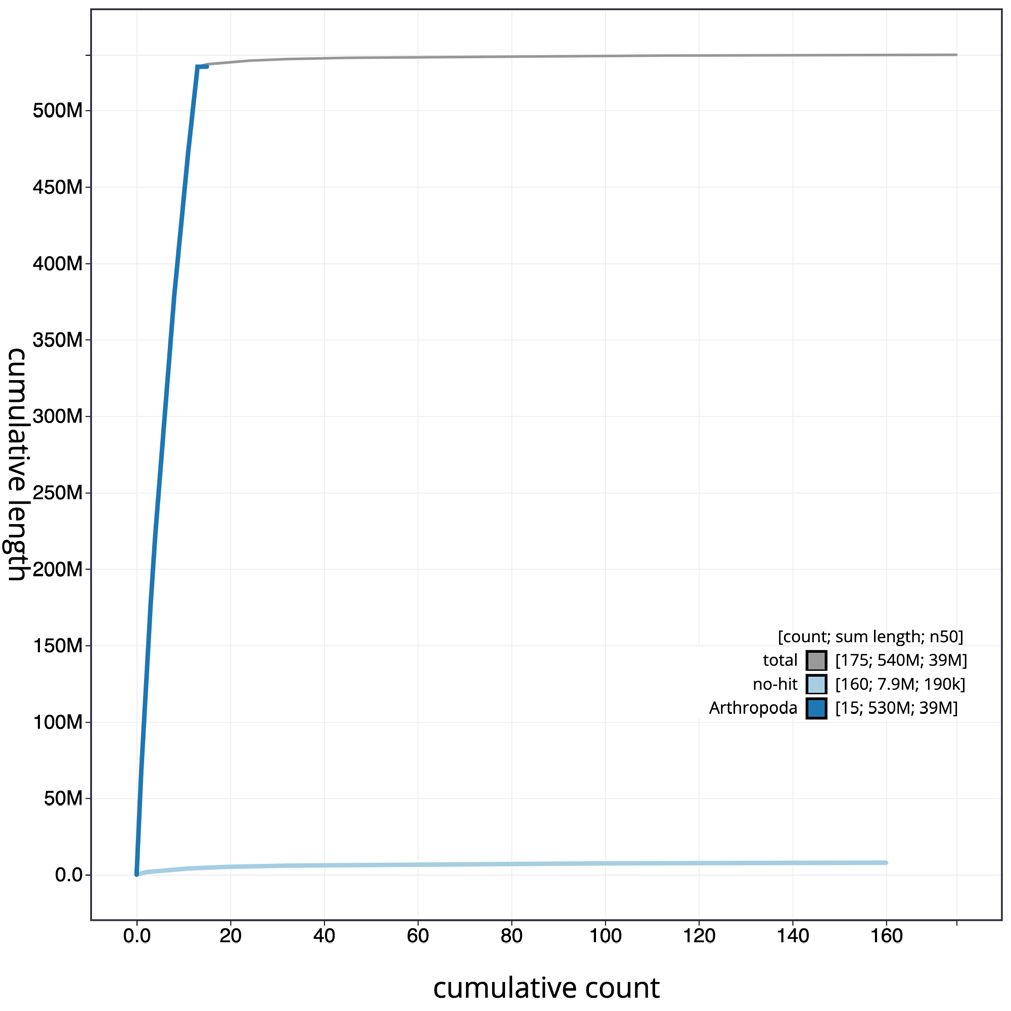
Genome assembly of *Leuctra nigra*, ipLeuNigr2.1: cumulative sequence. BlobToolKit cumulative sequence plot. The grey line shows cumulative length for all scaffolds. Coloured lines show cumulative lengths of scaffolds assigned to each phylum using the buscogenes taxrule. An interactive version of this figure is available at https://blobtoolkit.genomehubs.org/view/ipLeuNigr2.1/dataset/CAKOGZ01/cumulative.

**Figure 5.**
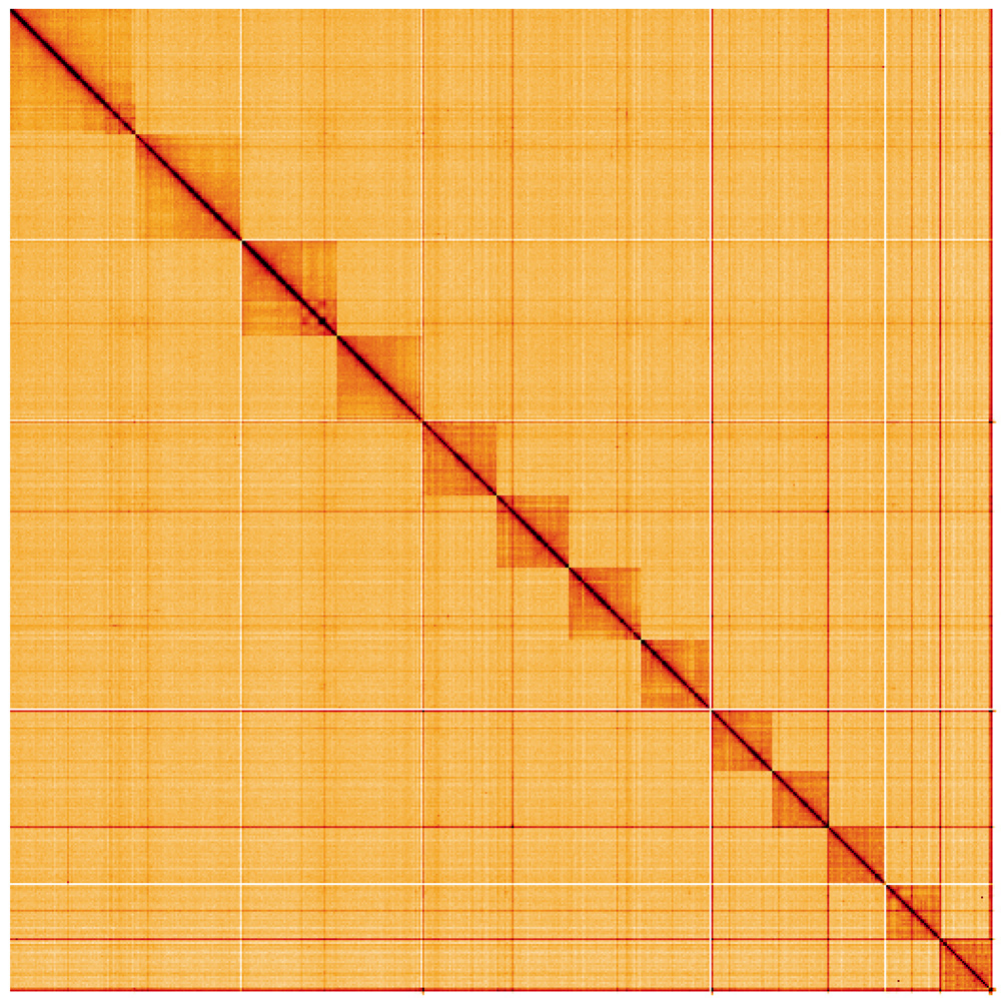
Genome assembly of *Leuctra nigra*, ipLeuNigr2.1: Hi-C contact map. Hi-C contact map of the ipLeuNigr2.1 assembly, visualised using HiGlass. Chromosomes are shown in order of size from left to right and top to bottom. An interactive version of this figure may be viewed at https://genome-note-higlass.tol.sanger.ac.uk/l/?d=XnHfqqyDSxilzwfKk4VUMw.

**Table 2.** Chromosomal pseudomolecules in the genome assembly of *Leuctra nigra*, ipLeuNigr2.

INSDC accession	Chromosome	Size (Mb)	GC%
OW203761.1	1	68.08	38.6
OW203762.1	2	57.02	38.5
OW203764.1	3	45.7	38.3
OW203765.1	4	40	38.2
OW203766.1	5	39.22	38.6
OW203767.1	6	39.16	38.2
OW203768.1	7	37.12	38.3
OW203769.1	8	33.46	38.2
OW203770.1	9	30.79	37.4
OW203771.1	10	30.19	37.5
OW203772.1	11	29.11	38.7
OW203773.1	12	26.82	37.7
OW203763.1	X	51.71	36.9
OW203774.1	MT	0.02	32.6

## Methods

### Sample acquisition and nucleic acid extraction

Two *Leuctra nigra* specimens (ipLeuNigr1 and ipLeuNigr2) were collected from River Taff Fawr, Garwnant, Wales, UK (latitude 51.808259, longitude –3.44498) using a kick-net on 19 March 2019. The specimens were collected and identified by Caleala Clifford (Natural Resources Wales) and snap-frozen in a dry shipper at the Natural History Museum, London.

DNA was extracted at the Tree of Life laboratory, Wellcome Sanger Institute (WSI). The ipLeuNigr2 sample was weighed and dissected on dry ice. The tissue was cryogenically disrupted to a fine powder using a Covaris cryoPREP Automated Dry Pulveriser, receiving multiple impacts. HMW DNA was sheared into an average fragment size of 12–20 kb in a Megaruptor 3 system with speed setting 30. Sheared DNA was purified by solid-phase reversible immobilisation using AMPure PB beads with a 1.8X ratio of beads to sample to remove the shorter fragments and concentrate the DNA sample. The concentration of the sheared and purified DNA was assessed using a Nanodrop spectrophotometer and Qubit Fluorometer and Qubit dsDNA High Sensitivity Assay kit. Fragment size distribution was evaluated by running the sample on the FemtoPulse system.

### Sequencing

Pacific Biosciences HiFi circular consensus DNA sequencing libraries were constructed according to the manufacturers’ instructions. DNA sequencing was performed by the Scientific Operations core at the WSI on Pacific Biosciences SEQUEL II (HiFi) instrument. Hi-C data were also generated from ipLeuNigr1 using the Arima v2 kit and sequenced on the HiSeq X Ten instrument.

### Genome assembly

Assembly was carried out with Hifiasm ( Cheng *et al.*, 2021) and haplotypic duplication was identified and removed with purge_dups ( Guan *et al.*, 2020). The assembly was then scaffolded with Hi-C data ( Rao *et al.*, 2014) using YaHS ( Zhou *et al.*, 2023). The assembly was checked for contamination and corrected as described previously ( Howe *et al.*, 2021). Manual curation was performed using HiGlass ( Kerpedjiev *et al.*, 2018) and Pretext ( Harry, 2022). The mitochondrial genome was assembled using MitoHiFi ( Uliano-Silva *et al.*, 2022), which performed annotation using MitoFinder ( Allio *et al.*, 2020). The genome was analysed and BUSCO scores generated within the BlobToolKit environment ( Challis *et al.*, 2020). Table 3 contains a list of all software tool versions used, where appropriate.

**Table 3.** Software tools and versions used.

Software tool	Version	Source
BlobToolKit	3.4.0	Challis *et al.*, 2020
Hifiasm	0.12	Cheng *et al.*, 2021
HiGlass	1.11.6	Kerpedjiev *et al.*, 2018
MitoHiFi	2	Uliano-Silva *et al.*, 2022
PretextView	0.2	Harry, 2022
purge_dups	1.2.3	Guan *et al.*, 2020
YaHS	yahs-1.1.91eebc2	Zhou *et al.*, 2023

### Ethics and compliance issues

The materials that have contributed to this genome note have been supplied by a Darwin Tree of Life Partner. The submission of materials by a Darwin Tree of Life Partner is subject to the Darwin Tree of Life Project Sampling Code of Practice. By agreeing with and signing up to the Sampling Code of Practice, the Darwin Tree of Life Partner agrees they will meet the legal and ethical requirements and standards set out within this document in respect of all samples acquired for, and supplied to, the Darwin Tree of Life Project. All efforts are undertaken to minimise the suffering of animals used for sequencing. Each transfer of samples is further undertaken according to a Research Collaboration Agreement or Material Transfer Agreement entered into by the Darwin Tree of Life Partner, Genome Research Limited (operating as the Wellcome Sanger Institute), and in some circumstances other Darwin Tree of Life collaborators.

## Data Availability

European Nucleotide Archive: *Leuctra nigra*. Accession number PRJEB50932; https://identifiers.org/ena.embl/PRJEB50932. ( Wellcome Sanger Institute, 2022) The genome sequence is released openly for reuse. The *Leuctra nigra* genome sequencing initiative is part of the Darwin Tree of Life (DToL) project. All raw sequence data and the assembly have been deposited in INSDC databases. The genome will be annotated using available RNA-Seq data and presented through the Ensembl pipeline at the European Bioinformatics Institute. Raw data and assembly accession identifiers are reported in Table 1.

## Author information

Members of the Natural History Museum Genome Acquisition Lab are listed here: https://doi.org/10.5281/zenodo.4790043.

Members of the Darwin Tree of Life Barcoding collective are listed here: https://doi.org/10.5281/zenodo.4893703.

Members of the Wellcome Sanger Institute Tree of Life programme are listed here: https://doi.org/10.5281/zenodo.4783585.

Members of Wellcome Sanger Institute Scientific Operations: DNA Pipelines collective are listed here: https://doi.org/10.5281/zenodo.4790455.

Members of the Tree of Life Core Informatics collective are listed here: https://doi.org/10.5281/zenodo.5013541.

Members of the Darwin Tree of Life Consortium are listed here: https://doi.org/10.5281/zenodo.4783558.

## References

[ref-1] Allio R Schomaker-Bastos A Romiguier J : MitoFinder: Efficient automated large‐scale extraction of mitogenomic data in target enrichment phylogenomics. *Mol Ecol Resour.* 2020;20(4):892–905. 10.1111/1755-0998.13160 32243090 PMC7497042

[ref-2] Baars JR Kelly-Quinn M : The Plecoptera of Irish freshwaters - species distribution, status and association with environmental parameters. *Report to the Heritage Council.* Reference no. 14525. [Preprint],2006.

[ref-3] Brinck P : Studies on Swedish Stoneflies (Plecoptera). *Opuscula Entomologica.* 1949;11:1–250.

[ref-4] Challis R Richards E Rajan J : BlobToolKit - interactive quality assessment of genome assemblies. *G3 (Bethesda).* 2020;10(4):1361–1374. 10.1534/g3.119.400908 32071071 PMC7144090

[ref-5] Cheng H Concepcion GT Feng X : Haplotype-resolved *de novo* assembly using phased assembly graphs with hifiasm. *Nat Methods.* 2021;18(2):170–175. 10.1038/s41592-020-01056-5 33526886 PMC7961889

[ref-6] Elliott JM : Temperature-induced changes in the life cycle of *Leuctra nigra* (Plecoptera: Leuctridae) from a Lake District stream. *Freshw Biol.* 1987;18(1):177–184. 10.1111/j.1365-2427.1987.tb01305.x

[ref-7] Feeley HB : The impact of mature conifer forest plantations on the hydrochemical and ecological quality of headwater streams in Ireland, with particular reference to episodic acidification. *National University of Ireland (UCD).* 2012.

[ref-8] Feeley HB Kelly-Quinn M : Re-examining the effects of episodic acidity on macroinvertebrates in small conifer-forested streams in Ireland and empirical evidence for biological recovery. *Biology and Environment: Proceedings of the Royal Irish Academy.* 2014;114B(3):205–218. 10.1353/bae.2014.0007

[ref-9] Friberg N Rebsdorf A Larsen SE : Effects of afforestation on acidity and invertebrates in Danish streams and implications for freshwater communities in Denmark. *Water, Air and Soil Pollution.* 1998;101:235–256. 10.1023/A:1004949203686

[ref-10] Graf W Lorenz AW De Figueroa JMT : Plecoptera: Volume 2.In: A. Schmidt-Kloiber and D. Hering (eds) *Distribution and Ecological Preferences of European Freshwater Organisms.* 2009;1–262.

[ref-11] Graf W Grasser U Weinzierl A : Plecoptera. Part III.In: O. Moog (ed.) *Fauna Aquatica Austriaca.* Wien: Wasserwirtschaftskataster, Bundesminterium für Land- und Forstwirtschaft, Umwelt und Wasserwirtschaft, 2002;17.

[ref-12] Guan D McCarthy SA Wood J : Identifying and removing haplotypic duplication in primary genome assemblies. *Bioinformatics.* 2020;36(9):2896–2898. 10.1093/bioinformatics/btaa025 31971576 PMC7203741

[ref-13] Harry E : PretextView (Paired REad TEXTure Viewer): A desktop application for viewing pretext contact maps. 2022; (Accessed: 19 October 2022). Reference Source

[ref-14] Henderson J Hildrew AG Townsend CR : Detritivorous stoneflies of an iron-rich stream: food and feeding.In: I.C. Campbell (ed.) *Mayflies and Stoneflies.* Dordrecht: Kluwer,1990;44:249–254. 10.1007/978-94-009-2397-3_29

[ref-15] Hildrew AG Townsend CR Henderson J : Interactions between Larval Size, Microdistribution and Substrate in the Stoneflies of an Iron-Rich Stream. *Oikos.* 1980;35(3):387–396. 10.2307/3544655

[ref-16] Howe K Chow W Collins J : Significantly improving the quality of genome assemblies through curation. *GigaScience.* Oxford University Press,2021;10(1):giaa153. 10.1093/gigascience/giaa153 33420778 PMC7794651

[ref-17] Hynes HBN : The taxonomy and ecology of the nymphs of British Plecoptera with notes on the adults and eggs. *Trans R Entomol Soc Lond.* 1941;91(10):459–557. 10.1111/j.1365-2311.1941.tb01039.x

[ref-18] Kerpedjiev P Abdennur N Lekschas F : HiGlass: Web-based visual exploration and analysis of genome interaction maps. *Genome Biol.* 2018;19(1):125. 10.1186/s13059-018-1486-1 30143029 PMC6109259

[ref-19] Lillehammer A : Stoneflies (Plecoptera) of Fennoscandia and Denmark. Leiden: Scandinavian Science Press Ltd,1988. Reference Source

[ref-20] Manni M Berkeley MR Seppey M : BUSCO Update: Novel and Streamlined Workflows along with Broader and Deeper Phylogenetic Coverage for Scoring of Eukaryotic, Prokaryotic, and Viral Genomes. *Mol Biol Evol.* 2021;38(10):4647–4654. 10.1093/molbev/msab199 34320186 PMC8476166

[ref-21] Murphy JF Davy-Bowker J McFarland B : A diagnostic biotic index for assessing acidity in sensitive streams in Britain. *Ecol Indic.* 2013;24:562–572. 10.1016/j.ecolind.2012.08.014

[ref-22] O’Connor JP Costello MJ : Leuctra nigra (Olivier) (Plecoptera: Leuctridae), a stonefly new to Ireland. *Entomologist's Gazette.* 1997;48:51–52.

[ref-23] Rao SSP Huntley MH Durand NC : A 3D map of the human genome at kilobase resolution reveals principles of chromatin looping. *Cell.* 2014;159(7):1665–80. 10.1016/j.cell.2014.11.021 25497547 PMC5635824

[ref-24] Ravizza C Vinçon G : Les Leuctridés (Plecoptera, Leuctridae) des Alpes. Mitteilungen der Schweizerischen Entomologischen Gesellschaft. *Bulletin de la Société Entomologique Suisse.* 1998;71:285–342.

[ref-25] Rhie A McCarthy SA Fedrigo O : Towards complete and error-free genome assemblies of all vertebrate species. *Nature.* 2021;592(7856):737–746. 10.1038/s41586-021-03451-0 33911273 PMC8081667

[ref-26] Stoner JH Gee AS Wade KR : The effects of acidification on the ecology of streams in the Upper Tywi catchment in west Wales. *Environmental Pollution Series A, Ecological and Biological.* 1984;35(2):125–157. 10.1016/0143-1471(84)90135-1

[ref-27] Thomsen AG Friberg N : Growth and emergence of the stonefly *Leuctra nigra* in coniferous forest streams with contrasting pH. *Freshw Biol.* 2002;47(6):1159–1172. 10.1046/j.1365-2427.2002.00827.x

[ref-28] Uliano-Silva M Ferreira JGRN Krasheninnikova K : MitoHiFi: a python pipeline for mitochondrial genome assembly from PacBio High Fidelity reads. *bioRxiv.* [Preprint],2022. 10.1101/2022.12.23.521667 PMC10354987 37464285

[ref-30] Wellcome Sanger Institute: The genome sequence of the black needle fly, *Leuctra nigra* (Olivier, 1811). European Nucleotide Archive. [dataset], accession number PRJEB50932,2022.

[ref-29] Zhou C McCarthy SA Durbin R : YaHS: yet another Hi-C scaffolding tool. *Bioinformatics.* Edited by C. Alkan,2023;39(1):btac808. 10.1093/bioinformatics/btac808 36525368 PMC9848053

