# Supplementary material for: The genome sequence of the black needle fly, Leuctra nigra (Olivier, 1811)
Source: Wellcome Open Res. 2023 Feb 22;8:95. [Version 1] doi: 10.12688/wellcomeopenres.19082.1 (PMC10280035; doi:10.12688/wellcomeopenres.19082.1)
Supplement: Peer review history

# Peer review: Reviewer response for version 1

**DOI:** 10.21956/wellcomeopenres.21157.r59713

**Published:** 2023-06-15

**Authors:** Shigeyuki Koshikawa, Takuma Niida

**Affiliations:** 1 Hokkaido University, Sapporo, Japan; 2 Graduate School of Environmental Science, Hokkaido University, Sapporo, Hokkaido Prefecture, Japan

This is a report on the sequencing of the genome sequence of a species of stonefly *Leuctra nigra*. It is a well-organized and concise report, especially from a technical point of view.

 In the Introduction, the authors described the habitat, but they should clearly state whether they are talking about nymphs or adults. Are we correct in understanding that the larvae are in water, such as streams, and the adults are on land? Are they nymphs or adults that are among the logs?

 >Genome sequence report

 >Manual assembly curation corrected 458 missing joins or mis-joins and

 >removed seven haplotypic duplications, reducing the scaffold number by

 >47.9%, and increasing the scaffold N50 by 4.62%

 In the "Genome sequence report", we wondered what information was used as the basis for manually fixing the assembly. Does it mean that there were references obtained from short reads? Do seven haplotypic duplications mean that there were seven pairs of similar sequences in the scaffolds?

 In Figure 5, we thought it would be easier to understand if the names (numbers) of the chromosomes were written on the vertical and horizontal axes.

 In the Methods, were nymphs or adults used in this study? Since the authors described that they were collected by kick-net, I assume they used nymphs collected from the water.

Are sufficient details of methods and materials provided to allow replication by others?

Yes

Is the rationale for creating the dataset(s) clearly described?

Yes

Are the datasets clearly presented in a useable and accessible format?

Yes

Are the protocols appropriate and is the work technically sound?

Yes

Reviewer Expertise:

Evolution and development of insects

We confirm that we have read this submission and believe that we have an appropriate level of expertise to confirm that it is of an acceptable scientific standard.

# Peer review: Reviewer response for version 1

**DOI:** 10.21956/wellcomeopenres.21157.r55268

**Published:** 2023-03-16

**Authors:** Maribet Gamboa

**Affiliations:** 1 Department of Ecology, Universidad Catolica de la Santisima Concepcion, Concepción, Bío Bío Region, Chile

The article mentioned the reconstruction of the *Leuctra nigra* genome based on one male specimen. The authors performed the Pacific Biosciences HiFi instrument and HiSeq XTen from two different haplotypes.

 The article mentioned methods and software used for replication purposes.

 I have minor observations/concerns:

- The authors don't specify why they chose specimen ipLeuNigr2 for the genome reconstruction.
- Why did the authors show ipLeuNigr2.1 on all figures and tables? There were different samples from ipLeuNigr2? I suggest keeping the same name to avoid confusion.
- The DNA quality and concentration from the chosen specimen should be mentioned.
- The second haplotype that the authors mentioned corresponds to ipLeuNigr1?
- How the contigs of the second haplotype helped the construction of the genome?

Are sufficient details of methods and materials provided to allow replication by others?

Yes

Is the rationale for creating the dataset(s) clearly described?

Yes

Are the datasets clearly presented in a useable and accessible format?

Yes

Are the protocols appropriate and is the work technically sound?

Yes

Reviewer Expertise:

Molecular biology, molecular ecology

I confirm that I have read this submission and believe that I have an appropriate level of expertise to confirm that it is of an acceptable scientific standard.
